# The Use of 2 e-Learning Modalities for Diabetes Education Using Facebook in 2 Cities of Argentina During the COVID-19 Pandemic: Qualitative Study

**DOI:** 10.2196/38862

**Published:** 2022-11-16

**Authors:** Daniela Luz Moyano, María Victoria Lopez, Ana Cavallo, Julia Patricia Candia, Aaron Kaen, Vilma Irazola, Andrea Beratarrechea

**Affiliations:** 1 Department of Research on Chronic Diseases Institute for Clinical Effectiveness and Health Policy Buenos Aires Argentina; 2 Ministry of Public Health of the Province of Chaco Resistencia Argentina; 3 Ministry of Health of the Province of La Rioja La Rioja Argentina

**Keywords:** COVID-19, social media, diabetes mellitus, public health, qualitative research, COVID-19 pandemic, teaching and learning settings, online learning, eHealth literacy

## Abstract

**Background:**

The COVID-19 pandemic and the confinement that was implemented in Argentina generated a need to implement innovative tools for the strengthening of diabetes care. Diabetes self-management education (DSME) is a core element of diabetes care; however, because of COVID-19 restrictions, in-person diabetes educational activities were suspended. Social networks have played an instrumental role in this context to provide DSME in 2 cities of Argentina and help persons with diabetes in their daily self-management.

**Objective:**

The aim of this study is to evaluate 2 diabetes education modalities (synchronous and asynchronous) using the social media platform Facebook through the content of posts on diabetes educational sessions in 2 cities of Argentina during the COVID-19 pandemic.

**Methods:**

In this qualitative study, we explored 2 modalities of e-learning (synchronous and asynchronous) for diabetes education that used the Facebook pages of public health institutions in Chaco and La Rioja, Argentina, in the context of confinement. Social media metrics and the content of the messages posted by users were analyzed.

**Results:**

A total of 332 messages were analyzed. We found that in the asynchronous modality, there was a higher number of visualizations, while in the synchronous modality, there were more posts and interactions between educators and users. We also observed that the number of views increased when primary care clinics were incorporated as disseminators, sharing educational videos from the sessions via social media. Positive aspects were observed in the posts, consisting of messages of thanks and, to a lesser extent, reaffirmations, reflections or personal experiences, and consultations related to the subject treated. Another relevant finding was that the educator/moderator role had a greater presence in the synchronous modality, where posts were based on motivation for participation, help to resolve connectivity problems, and answers to specific user queries.

**Conclusions:**

Our findings show positive contributions of an educational intervention for diabetes care using the social media platform Facebook in the context of the COVID-19 pandemic. Although each modality (synchronous vs asynchronous) could have differential and particular advantages, we believe that these strategies have potential to be replicated and adapted to other contexts. However, more documented experiences are needed to explore their sustainability and long-term impact from the users' perspective.

## Introduction

According to data from the International Diabetes Federation, there were 1 in 11 (32 million) adults with diabetes [1] in South and Central America in 2021. In Argentina, the prevalence of diabetes significantly increased from 8.4% to 12.7%, corresponding to a 51% rise during the 2005-2018 period [2].

Since the beginning of the COVID-19 pandemic, operational guidelines for maintaining essential health services focused on increasing awareness of persons with diabetes about their susceptibility to COVID-19 and ways to reduce transmission, increasing home supplies of medication, and modifying routine clinical visits to videocalls or phone calls, telehealth, or email [3,4]. As part of the health care system reorganization, the Ministry of Health implemented a telehealth network in the country [5] but it was mainly focused on the diagnosis and follow-up of persons with COVID-19 and was not widely implemented in public primary care clinics to provide care for underserved persons with diabetes or other chronic care conditions.

Persons with diabetes in Argentina faced different barriers to access to chronic care, which included total confinement, health care system reorganization, and the sense among patients that seeking care implied an increased risk of COVID-19 infection. In addition, the adoption of healthy habits was difficult to achieve due to confinement [6].

Diabetes self-management education (DSME) is a critical element of diabetes care as it improves patient outcomes and quality of life and prevents complications [7]. Prepandemic, only 2 of 10 patients had ever participated in either a DSME session or a workshop, although it is included as a core care element by the diabetes clinical practice guidelines (DCPG) [8].

Social media use increased during the pandemic as a new dynamic way to share information in a rapid and accessible way. In Argentina, in 2020, nearly 85.5% of people aged 4 years or more had access to and used the internet [9], and 9 of 10 internet users were on social media, Facebook being 1 of the most frequently used platforms (86%) [10]. Social media posts and reliable information can promote health and well-being, health awareness, and preventive behaviors [11,12], helping acknowledge chronic conditions and guiding the public on how to manage and contain them.

The use of social media for diabetes self-care is still incipient [13]. Although some studies have shown that social media has beneficial effects in diabetes knowledge, attitudes, and self-care activities, results regarding patient outcomes are still mixed [14-16], especially for type 2 diabetes. Because of the lockdown and restrictions due to the COVID-19 pandemic in the country [17], we implemented social media interventions to provide DSME in 2 cities of Argentina to help persons with diabetes in their daily self-management.

The aim of this study was to evaluate 2 diabetes education modalities (synchronous and asynchronous) using the social media platform Facebook through the content of posts on diabetes educational sessions in 2 cities of Argentina during the COVID-19 pandemic.

## Methods

### Design and Study Setting

A qualitative approach was used to explore an intervention to provide diabetes education adapted to the context of confinement during the first year of the COVID-19 pandemic in the provinces of La Rioja and Chaco, Argentina [18,19], via the social network Facebook.

The Facebook public figure pages of 2 health institutions, the Diabetes and Nutrition Service of the Julio C. Perrando Hospital in Chaco and the Directorate of Chronic Non-Communicable Diseases in La Rioja, were used to disseminate educational sessions. This initiative was carried out jointly by the Institute for Clinical Effectiveness and Health Policy and the Diabetes Programs of the provinces of Chaco and La Rioja.

### Structure of the Diabetes Education Intervention

Diabetes educational sessions were conducted using 2 modalities, synchronous and asynchronous, using the Facebook social media platform. Diabetes educator–led sessions included the topics listed in Table 1.

For the transmission and dissemination of the sessions, we used the Facebook pages of 2 health institutions in Chaco and La Rioja. In the latter case, educational sessions were also disseminated through the Facebook pages of 7 primary health care clinics.

Diabetes educational sessions were directly aimed at users of both pages and open to anyone interested in diabetes.

**Table 1:** Topics and content of the sessions (N=9) according to the modality implemented.

Topics	Content examples	Asynchronous modality sessions (n=4, 44%), n (%)	Synchronous modality sessions (n=5, 56%), n (%)
Diabetes care during confinement	Diabetes and obesity, prevention during the pandemic, hand hygiene practices, cleaning and disinfection to reduce transmission of COVID-19, and general recommendations for healthy eating, mental health, and physical activity	1 (25)	1 (20)
Diabetic foot	Warning signs, foot examination, foot care, red flags for consultation, treatment, and complications	1 (25)	1 (20)
Insulin management	Facts and myths about insulin therapy, barriers to insulin use, beliefs, safe and effective use of insulin, and psychological aspects in insulin therapy during confinement	1 (25)	1 (20)
Healthy eating and diabetes	Recommendations of the Dietary Guidelines for the Argentine Population, homemade healthy food and portion control, meal preparation, and facts and myths about food in diabetes management	1 (25)	N/A^a^
Physical activity and diabetes	Physical activity goals during confinement, physical activity recommendations in type 1 and type 2 diabetes, physical activity prescription, nutrition in physical activity, and management of hypoglycemia	N/A	1 (20)
Mental health, diet, and diabetes	How emotions can affect us, food management at home, role of hunger and satiety in weight management, food craving, dealing with emotions, importance of sleep, psychological support, proper nutrition in the pandemic, and dietary recommendations	N/A	1 (20)

^a^N/A: not applicable.

#### Synchronous Modality

This modality was implemented in the province of Chaco. For this strategy, in addition to Facebook, we used Streamyard [20], a live streaming platform that allowed multiple diabetes educators to participate in the streaming. The team that conducted the workshop comprised health educators and a moderator. To market the activity, we designed an informative flyer and posted it on the Facebook page. We also shared this post through WhatsApp groups with persons with diabetes and health care providers from primary care clinics a week prior to the event. The educational sessions were held between July 30 and October 29, 2020 (Table 1).

Sessions took between 45 and 60 minutes and were structured in 3 parts. In the first part, the moderator introduced the diabetes educators and explained the activity. In the second part, the diabetes educators presented a topic included in the educational diabetes curriculum of the DCPG (Table 1), which was adapted to the context [8]. This was followed by a question-and-answer segment, during which the diabetes educators responded to the attendees’ questions collected by the moderator. Finally, the workshop live-streaming recording was posted on the Diabetes and Nutrition Service of the Julio C. Perrando Hospital Facebook pages, and diabetes educators got an opportunity to review and answer further questions and comments that were posted by the audience afterward.

#### Asynchronous Modality

The asynchronous modality was developed in the province of La Rioja, and educational sessions were implemented from August 31 to November 30, 2020. Some of the sessions were developed using the educators’ computers and others using a mobile phone and were recorded using the Zoom platform [21], a cloud-based video communications app. The diabetes educator, a health care professional, recorded a 30-minute session on a topic of interest (Table 1). The marketing of these sessions was carried out through informative flyers that were posted on Facebook and shared on WhatsApp groups a week prior to the event. On the date of the educational session, the video was released on the host’s Facebook page. Afterward, the diabetes educator also functioned as a moderator, reviewing and responding to the participants’ comments, questions, or doubts posted in the video. In addition to using this modality, we shared the link with 7 public primary care clinics involved in the diabetes program that had Facebook pages. Primary care clinics, through their social networks, became amplifiers.

### Units of Analysis

The units of analysis were the messages posted during the synchronous and asynchronous sessions implemented.

In addition, we analyzed social media metrics, such as impression (number of views) and engagement (eg likes, comments, and shares) in both modalities [22]. These data were used to contextualize the qualitative results.

### Dimensions and Categories of Analysis

#### Dimension: Social Media Metrics

This dimension had the following categories [22]:

- Category: Impression is the number of times the content was on a person’s screen (indicator: number of views).
- Category: Engagement relates to the number of interactions with a post, and it is important when you want an engaged audience to take some form of action after reading your post (indicators: number of likes, number of comments, and number of shares).

#### Dimension: Role of the Moderator

From a pedagogical point of view, the role of the moderator in information and communication technology (ICT)–mediated education acquired a crucial role; sometimes, the moderator was also the expert/educator, although these could also be separate roles [23].

#### Dimension: Role of the Participant

Participants were the Facebook account audience of the health institutions. Based on the analysis of the content of the posts made in each educational session, an analytical framework was developed. Four subdimensions were explored. Some similar dimensions were considered from a previous study [24].

**Figure 1:**
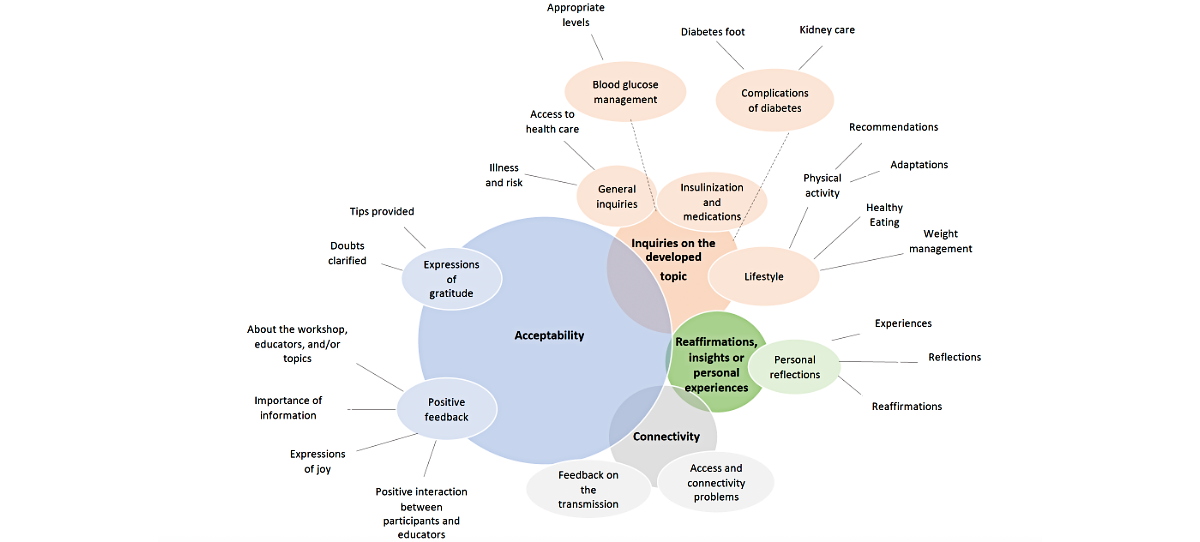
Emerging framework for the role of the participant.

From the analysis of the messages posted, we developed a qualitative framework (Figure 1). We included all the dimensions that emerged from the collected data regardless of the number of times they appeared. The framework reflects the preponderance that each principal subdimension had according to the frequency at which each subdimension appeared.

### Data Collection

Data collection was carried out by members of the research team through a systematic search for information on the Facebook pages of the participating institutions. It was based on the posted messages, likes, and views.

Quantitative data on social media metrics were collected weekly, including information from September 29 to December 29, 2020. Both qualitative and quantitative information fed the online matrix developed for this study.

### Data Analysis

Quantitative data from social metrics indicators were analyzed using means (SDs) per educational session. Analyses were conducted using Stata 14.0 [25].

We conducted an active analysis on Facebook [18]. All wall-posted content during the sessions was analyzed through content analysis [26]. In the first stage, a process of familiarization with the information was carried out. The research team was part of the audience (without active participation) to understand the context. The second stage consisted of a first analysis of the data set before coding by a qualitative researcher when a first approximation to the dimensions and categories was elaborated.

In the third stage, data were extracted manually, a content review of the input was generated, and a new, in-depth review of all units of analysis was conducted. Subsequently, texts, visual elements, phrases, words, and emoticons were transcribed, classified, and coded. From the selected dimensions, interpretive and emerging categories were generated. To increase the internal validity of the study, 2 researchers carried out triangulation [27].

The written transcripts were entered into ATLAS.ti version 6.2 [28] and combined with the manual technique of information coding. As part of the analysis, representative direct quotations were selected and included in this paper to illustrate our findings. Messages and social metrics indicator data were reviewed at least twice by the same reviewer to improve the data quality of this study.

### Ethical Considerations

Ethical approval was obtained in Chaco through the Hospital Pediátrico Avelino Castelán (Avelino Castelán Paediatric Hospital) and in La Rioja through the Consejo Provincial de Ética de las Investigaciones en Salud/CoPeis (Provincial Council of Health Research Ethics/CoPeis) (No 001). All data extracted were anonymized before being analyzed.

## Results

In total, 9 educational sessions were conducted, 5 (56%) through the synchronous modality and 4 (44%) through the asynchronous modality.

### Social Metrics in the Synchronous Modality

In the impression category, the mean number of views per educational session was 936.4 (SD 327.0). The total number of views of all educational sessions was 4682.

In the engagement category, the mean number of likes, comments, and shares per educational session were 51.2 (SD 10.9), 49.4 (SD 18.1), and 18.0 (SD 9.7), respectively.

### Social Metrics in the Asynchronous Modality

In the impression category, the overall mean number of views was 1618.3 (SD 728.8) and the total number of views of all educational sessions was 6473.

In the engagement category, the mean number of likes, comments, and shares per educational session were 37.8 (SD 6.7), 13.8 (SD 19.7), and 36.8 (SD 14.9), respectively.

In addition to this modality, a dissemination/amplification process was also carried out through the Facebook pages of 7 primary care clinics. There was a mean increase of 109.3% (1618.2 to 3386.7) in the number of views, 238.4% (37.7 to 127. 7) in the number of likes, 52.7% (13.7 to 21) in the number of comments, and 132.0% (36.7 to 85.2) in the number of shares.

### Post Content Analysis

A total of 332 wall posts corresponding to the 9 educational sessions were analyzed. Participant comments accounted for 90.1% (299/332), while educators/moderators’ comments corresponded to 9.9% of all comments over the study period.

The results obtained from the analysis of the content of the comments posted are presented next and were organized into 2 main dimensions: the moderator role and the participant role. In the first case, since differences were found in emerging dimensions, the results are presented separately for the synchronous and asynchronous modalities, while we grouped the results for the participant role as no significant differences were observed during this process.

#### Moderator Role

In total, 4 subdimensions of post analysis emerged from this study: motivation for participation, resolution of connectivity problems, answers to specific user queries, and feedback to the expert/educator.

In the synchronous modality, there was a greater presence of the moderator's role and most of the messages were based on the “motivation for participation” subdimension. There were also messages with guidance for attendees to solve “connectivity problems,” although these were few. Some of the messages were as follows:

Share myths and beliefs you know about Insulin and its use.

Yes, of course. Leave your doubts and comments and we will share them.

We look forward to your questions and concerns!!!

Hello (attendee’s name), try logging out and in again.

Good afternoon, it's probably your internet connection because it looks and sounds fine.

Messages from the moderator included in the “answers to specific user queries” subdimension (eg, facilitate appointment scheduling to the hospital, respond to queries on the content, market upcoming workshops) were observed in both synchronous and asynchronous modalities, although less frequently in the latter.

In addition, during the educational sessions, the expert/educator provided feedback to address participants’ concerns. Some messages related to this dimension were as follows:

That should be answered on an individualized basis; you should schedule an in-person visit.

(Attendee's name) definitely not. You have to look for other strategies to cope with stress, never food.

Yes, diabetes can produce that dryness in your feet, and it is important that you moisturize them and control them every day, because if a lesion appears, you have to make the medical consultation immediately...

#### Participant Role

Messages posted were short texts (256/299, 85.6%) with or without the inclusion of emoticons. In both modalities, 30 (10.0%) of the 299 messages were based only on emoticons/GIFs, such as clapping hands or smiley faces expressing joy. Some of the messages posted were participants’ medical concerns that were later addressed during the educational sessions.

We did not find posts of images, videos, or links to other web pages. Although they were infrequent, tags to other Facebook users were observed that included either other people or primary care clinics (13/299, 4.3%). Table 2 presents the results of the analysis of the 4 subdimensions and their respective emerging categories from the participant role. The following new emerging subdimensions were identified: (1) inquiries on the topic developed, (2) connectivity, (3) acceptability, and (4) reaffirmations, insights, or personal experiences.

**Table 2:** Subdimensions, categories, descriptions, and illustrative messages on the message profiles in the synchronous and asynchronous modalities.

Subdimension and category			Description		Illustrative messages
**Inquiries on the developed topic**					
	General inquiries	Messages about diabetes and COVID-19 risk or access to health care (inquiry about appointments)		“What is the reason that we diabetes sufferers are a risk group?” “...Very good!!! I am diabetic. Which are the consulting days?”	
	Insulinization and medications	Messages about medication, especially insulin (focused on the initiation of insulin use)		“I would like to know why correction insulin sometimes doesn't work for me; does it make a difference if the patient starts insulin treatment directly?”	
	Blood glucose management	Messages about blood glucose and its appropriate levels		“Good afternoon. What can I do? I have a blood glucose value of 110 and I would like to lower it. I am not yet diagnosed as diabetic”	
	Complications of diabetes	Messages related to chronic complications, kidney care, uremia, and diabetes foot		“I would like to make a consultation. I have type 1 diabetes and chronic kidney disease on continuous ambulatory peritoneal dialysis. My feet are too dry and are have brown stains. Can it be because of increasing urea because my A1c is below 6” “Hello, I am a type 2 diabetic. My feet are dry and itchy at night. They don't have sores or lesions”	
	Lifestyle	Messages about healthy eating (eg, the use of diet products, foods linked to anxiety, nuts, whole foods) and weight management, physical activity (recommended time, exercise that they can do at home, routines adapted to specific groups)		“My mother asks: to satiate the sugar craving, are nuts or cereals with sweetener useful? ”What foods do you recommend for sugar craving when you are anxious?“ ”An exercise routine to do at home“ ”Regarding physical activity, e.g., walking. How long is recommended?“	
**Connectivity**					
	Access and connectivity problems	Comments on problems to access, video, connectivity, or sound		”I can't see the whole picture“ ”I can't hear well“ ”Many people do not know how to connect to the diabetes talk“	
	Feedback on transmission	Messages related to watching educational sessions at a later time and indicating that it is heard well, whether they can post queries, and the frequency in which that the workshops will be held		“Hi, we are watching the recorded educational session…” “I welcome this initiative and also to have more channels of communication. The Whastapp group we have is daily and helps a lot. I wanted to know how often we are going to see you and get in touch through Facebook and through live broadcasts”	
**Acceptability**					
	Expressions of gratitude	Messages with thank-you comments for the tips provided and doubts clarified		”Thank you for everything and especially for offering online care. Priceless advice“ “Very interesting!!! Thank you for educating us”	
	Positive feedback	Messages with positive feedback about the workshop, educators, or topics addressed		”I liked it very much, very useful for those of us who live far from the Capital city and do not have a diabetologist...thank you“ ”Very interesting, exposing what happens to us daily helps us to understand it better and to be able to focus in another way“ “Very good explanation. You help us a lot. I’m looking forward to the next session! “Congratulations to the two speakers, very clear, and using simple language without loosing scientific rigor”	
**Reaffirmations, insights, or personal experiences**					
	Personal reflections	Comments of users on their own experiences, reflections, or reaffirmations about what was presented in the session (eg, importance of physical activity, diet, and emotions)		“It was hard for me to get out of this, but I am achieving it little by little and I started walking, cycling and dancing zumba and when the pandemic started, I felt like eating all the time, but I started with my diet. Thanks to the group and the doctor and (says the name) for helping me” “It is important that we can learn for ourselves and teach our children to express our emotions”, ... we can always improve” “… exposing what happens to us daily helps us to understand it better and be able to approach the disease in a different way”	

#### Subdimension: Inquiries on the Developed Topic

The majority of the messages posted in this subdimension were not directly related to the topic presented during the educational session but questions and concerns participants had regarding diabetes care (Table 2).

#### Subdimension: Connectivity

A few messages were posted under the “connectivity problems and feedback on transmission” category, which expressed connectivity difficulties related to video or audio problems mainly in the synchronous modality (Table 2).

#### Subdimension: Acceptability

Under the “expressions of gratitude” category, we observed that a large portion of the messages posted during the sessions (in both synchronous and asynchronous modalities) were thank-you messages and positive feedback. Most of these messages were accompanied by emoticons/GIFs (Table 2).

In some cases, the messages consisted of joy or satisfaction or both with the educational session and expressions of gratitude toward the educators.

Under the “positive feedback” category, some of the messages expressed the importance of and the help provided by the educational sessions and asked about the possibility of continuing with these activities. In some cases, positive interaction was observed among participants and between participants and educators.

There were no negative messages or expressions of discomfort in either of the 2 modalities under analysis.

#### Subdimension: Reaffirmations, Insights, or Personal Experiences

To a lesser extent and only in the synchronous modality, comments related to short stories about the participants’ own individual experiences, reflections, or reaffirmations related to the content of the educational sessions were observed. The statements were based on sharing the positive changes made to manage diabetes (Table 2).

## Discussion

### Principal Findings

Our findings showed that both modalities (synchronous and asynchronous) have their own advantages and limitations. During the implementation of the synchronous modality, we observed a greater interaction between educators and participants; the educators played a proactive role by answering questions in real time, motivating the interaction, and helping participants solve technical problems that occurred during livestream educational sessions. These particular advantages of synchronous e-learning sessions were highlighted in other studies conducted with students during the COVID-19 pandemic [29,30]. A limitation found in implementing this modality was the need to have access to good internet connectivity during live streaming. To ensure equitable access using this modality, it is important to provide asynchronous options by posting the video after live streaming in the social media platform. Connectivity barriers were also reported in other studies when using the synchronous modality [29,31].

Asynchronous e-learning provides an opportunity for learning at a time and place that are convenient and not competing with personal time or work demands [32]. This could be a possible explanation for a higher number of visualizations of the videos posted in the asynchronous modality compared to the synchronous modality, which implied an increased social reach.

In both modalities, although more frequently in the synchronous modality, participants interacted mainly with educators by posting questions and messages regarding diabetes care and COVID-19 that in another context would have been addressed during a clinic consultation with health care professionals.

Although social media platforms foster interaction between users, peer interaction was infrequent during synchronous and asynchronous educational sessions. The Association of Diabetes Care & Education Specialists pointed out the clinical, behavioral, psychological, and educational benefits of online peer support [33]. In this sense, strategies to foster peer exchange and connection through social media need to be included as part of the educational intervention.

An interesting finding was that sharing the link to primary care clinics’ social media pages increased the social media reach of the proposed educational intervention as well as users’ engagement.

We found that participants posted messages related to the emotional effects of living with diabetes in the context of the pandemic and the impact it had on their self-care activities. These findings are in line with other studies that highlighted the emotional impact the pandemic had on people with chronic conditions [34,35].

In both modalities, a large proportion of the messages posted expressed positive perceptions and experienced acceptability of the social media intervention to educate and support persons with diabetes and their families in a context of confinement.

To the best of our knowledge, this is the first study to analyze the content of the posts of 2 diabetes education modalities using the Facebook social media platform during the COVID-19 pandemic. In agreement with our findings, Thomas et al [36] found that the use of social media platforms (Twitter and YouTube) constituted a reliable method of education for people with diabetes during the COVID-19 pandemic. A distinctive fact that arises from our work is that we added content analysis of the posts to the social media metrics analysis.

A previous local experience before the COVID-19 pandemic showed that educational strategies based on information technologies aimed at people with diabetes in a context of vulnerability had positive contributions in different dimensions of integral health [37].

However, a greater body of knowledge is still needed regarding the use of social media to provide diabetes education in low- and middle-income countries (LMICs). A scoping review published before the pandemic by Dol et al [38] identified 414 papers that used social media for research purposes, with 1.2% (5/414) of the first authors based in LMICs and only 3 studies focusing on patient education and care. In the same line, Hagg et al [39] explored the state of literature on the use of social media for health purposes in LMICs and found that 28 of 40 papers focused on health education. However, education campaigns using social media included themes such as tobacco use, support for HIV patients under treatment, sexual health behaviours, Middle East respiratory syndrome (MERS), malaria, and dengue but not chronic diseases.

Some authors stated that telehealth and other digital media were used safely and effectively for diabetes training and education for persons with diabetes during the pandemic [36,40,41].

The use of social networks in Latin America and the Caribbean has a great reach, with Facebook being 1 of the most popular social networks [42] and granting unique communication capabilities that can serve different educational purposes. It appears to be a promising technique for promoting health and wellness [43] and helping propagate health information [44].

However, studies based on social networks, and even more so for chronic diseases and diabetes education, are still currently limited and robust evidence is not yet available [45]. More work is needed in this field postpandemic.

### Strengths and Limitations

One of the main strengths of our study was the qualitative analysis of a significant number of posts. From the analysis of the content of the posts, we developed a framework (emerging dimensions) to theorize about the contributions the use of social media for diabetes education had for the users in the context of the COVID-19 pandemic and the confinement. It would be a helpful next step to validate this framework in other contexts. Another strength to highlight is that we explored a topic with little precedent in the current literature in an LMIC that could be useful for present and future virtual educational interventions.

There are some limitations to be considered in our study. The study was conducted using public Facebook accounts, so we did not have information regarding the users who participated in educational sessions. In Argentina, there are 33.8 million people who access Facebook, most are between 25 and 34 years old, and, therefore, the older population would be excluded from diabetes education provision [46]. We are also aware that there may be a selection bias of the population under analysis as messages posted might correspond to adherent persons or people who can be considered more aware of the health topics covered. Another limitation is derived from the qualitative research itself, such as the lack of generalizability of the results to a nonpandemic context and the use of other social networks not reached in the study that might be more widely used in the future.

### Implications for Practice

Based on the results and the analysis carried out, the following general recommendations are proposed for health teams and decision makers when designing and implementing educational strategies for diabetes using social media:

- When designing e-learning strategies using social media, it is necessary to analyze what type of modality best suits the needs of the target population and consider access to social networks, the type of social network, and connectivity in the target population.
- It is important to address the level of support needed by diabetes educators to plan educational sessions and develop educational materials for social networks. A synchronous e-learning modality needs more interaction and exchange between educators and users. Educators have to provide timely support to users so they can follow the educational sessions. If there are barriers to technologies or to high-speed internet, resulting in difficulties to participate, an asynchronous flexible modality should be proposed.
- Regardless of the modality chosen, a monitoring process of the posts is necessary to provide answers to users and avoid possible situations of misinformation.
- Strategies to motivate peer-to-peer interaction should be included.
- Social media reach can be enhanced by the incorporation of the social networks of health centers as disseminating/amplifying channels.
- Both modalities may have differential advantages, so a combination of synchronous and asynchronous strategies may be optimal in the framework of e-learning based on an analysis of the local context, needs, and barriers of the communities.

### Conclusion

The study findings show the positive contribution of implementing e-learning strategies using both synchronous and asynchronous modalities through a social media platform in the context of the COVID-19 pandemic for diabetes care support and education.

The differential potential of each modality (synchronous or asynchronous) needs, barriers, and particularities of the users should be considered.

Further studies are needed to explore their sustainability and impact in the medium and long term and within other implementation contexts, including barriers and facilitators.

## Abbreviations

DCPG: diabetes clinical practice guidelines
DSME: diabetes self-management education
LMIC: low- and middle-income country
